# Effectiveness of Bilayer Scaffold Containing Chitosan/Gelatin/Diclofenac and Bovine Hydroxyapatite on Cartilage/Subchondral Regeneration in Rabbit Joint Defect Models

**DOI:** 10.1155/2024/6987676

**Published:** 2024-09-26

**Authors:** Andhi Suyatno, Wa O. Nurfinti, Chika P. A. Kusuma, Yusuf A. Pratama, Chrismawan Ardianto, Samirah Samirah, Erreza Rahadiansyah, Junaidi Khotib, Aniek S. Budiatin

**Affiliations:** ^1^ Faculty of Pharmacy Universitas Airlangga, Surabaya 60115, Indonesia; ^2^ Department of Pharmacy Practice Faculty of Pharmacy Universitas Airlangga, Surabaya 60115, Indonesia; ^3^ Department of Orthopaedics and Traumatology Faculty of Medicine Universitas Airlangga, Surabaya 60131, Indonesia

## Abstract

Subchondral defects are often caused by trauma involving cartilage damage, leading to subsequent damage to the underlying bone, specifically the subchondral region. Bilayer scaffolds made from biomaterials, such as bovine hydroxyapatite, possess biocompatible and biodegradable properties that mimic the natural environmental conditions of target tissues so that they can support the formation of new tissues. On the other side, diclofenac as an anti-inflammatory drug potentiates to inhibit the inflammatory excess regarding the damage. This study aims to study the effectiveness of diclofenac scaffold to rabbit joint defect model. The scaffold was implanted in the rabbit femoral trochlear bone hole, which had a diameter of 5 mm and a depth of 4 mm. After 28 days of intervention, the animals were examined using macroscopic evaluation, hematoxylin-eosin (HE) staining, and immunohistochemistry (IHC) for type I collagen and type II collagen. Subsequently, the cartilage was evaluated using the International Cartilage Repair Society (ICRS) scoring system. The macroscopic ICRS scores were significantly higher (*p* < 0.05) in the bilayer scaffold implantation group compared to the monolayer scaffold and control groups. Histological ICRS scores were also significantly higher (*p* < 0.05) in the bilayer scaffold group compared to the control group. Type II collagen expression was higher (*p* < 0.05) in the bilayer scaffold group compared to the monolayer scaffold and control groups, although type I collagen expression was lower in comparison. In conclusion, this research suggests that the diclofenac-loaded bilayer scaffold effectively enhances cartilage and subchondral bone regeneration.

## 1. Introduction

Cartilage damage in the knee joint is caused by disease, old age, or osteochondral defects (OCD) that can decrease the quality of life of the patient. OCD is often due to trauma, which is usually the result of a sports injury or an accident. Many tissue engineering biomaterials have been developed and mostly evaluated in preclinical animals with standard OCD models by ignoring the inflammatory environment [1]. Cartilage is a tissue that lacks blood supply and reparative potential, so those characteristics make the cartilage difficult to be repaired [2]. In addition, the cartilage and subchondral bone have different microstructures and physiological functions. Therefore, the treatment of OCD remains a significant clinical challenge [3].

Cartilage-subchondral tissue engineering is essential in joint replacement in repairing cartilage-subchondral dysfunction, so to cope with it, a bone graft shaped as a scaffold has compositional similarities to a subchondral-cartilage regeneration intervention [3]. Three-dimensional (3D) bioprinting has now been considered a promising technique of cartilaginous tissue engineering that can replace damaged or missing cartilage with 3D-printed biological materials [2]. Scaffold plays an important role in many regenerative therapies with a tissue design approach that is capable of restoring and enhancing tissue function by providing a suitable substrate for cells to grow, reproduce, and differentiate [4].

Articular cartilage, subchondral bone, and calcified cartilage are the three joint constituents that form the biocomposite unit, defined as the osteochondral unit (OC), having the unique ability to transfer loads during load-bearing and joint movement. The cartilage and subchondral bone have different compositions, structures, biochemistry, and biomechanics [5], so a bilayer scaffold can be made with different compositions for each layer [6]. There are two different layers of bilayer scaffolds that resemble the cartilage and subchondral bone. The development of bilayer scaffolds in OC tissue engineering that simultaneously regenerate the cartilage and subchondral bone has been considered a desirable strategy. In addition, the bilayer scaffold can also maintain stability and good mechanical properties in the early stages of implantation. A bilayer scaffold can effectively avoid movement and shift of the cartilage layer [7].

Scaffolds can be made from both natural and synthetic polymers. Scaffolds that are made from natural materials are obtained from substances derived from microorganisms as well as from synthetic scaffolds made through chemical processes [8]. Natural and synthetic scaffolds have shown to contribute to the development of the cartilage [9]. Compared to synthetic polymers, natural polymers such as collagen, fibrin, silk protein, chitosan (Chi), and hyaluronic acid generally have better biocompatibility [10]. In the previous research from Sidney et al. [11], scaffold containing gelatin, chitosan, and diclofenac sodium has proven to accelerate cartilage regeneration in woven bone white rats *Rattus norvegicus* at the implantation site after 28 days. In Song et al.'s and Zhao et al.'s [12, 13] studies, animals which were given gelatin scaffold with sodium diclofenac could decrease the mRNA expression of CD3 and CD68 significantly than the control group. Sodium diclofenac has shown to inhibit PGE2 and nitrite in *in vitro* tests. PGE2 and nitrite are inflammatory mediators in response to inflammation. Diclofenac inhibits PGE2 production through the cyclooxygenase-2 (COX-2) pathway. The release of diclofenac from the scaffold has the potential to reduce PGE2 production, thereby reducing the acute inflammatory response [11].

In this research, bilayer scaffold was made with a composition of gelatin (Gel)-chitosan (Chi), hydroxyapatite (HA), and diclofenac sodium (ND). The HA that will be used comes from the bovine bone and is called bovine hydroxyapatite (BHA). The BHA used in this formula has several advantages compared to synthetic HA, namely, higher porosity (80%), synthetic HA (67%), and carbonate groups, which can increase osteoblast proliferation, resulting in rapid bone formation [14]. The high calcium availability provided by BHA as a delivery system accelerates the bone remodeling process. BHA extracted from bovine bones and current extraction methods have been proven to produce high amounts of pure hydroxyapatite and reduce the use of hazardous reagents [15]. Another advantage is that the calcium and phosphate (Ca/P) ratio of BHA of 1.62 ± 0.09 is closer to the Ca/P ratio of human bones of ±1.67 when compared to the Ca/P ratio of synthetic HA of 1.58 ± 0.15 [16]. Therefore, the addition of BHA to the bilayer scaffold is expected to accelerate the repair of subchondral defects compared to previous studies.

This paper presents knowledge about the comparison of the rate of cartilage repair between the control group, the Chi-Gel-BHA scaffold, and the bilayer. The improvement of cartilage was seen from macroscopic chondrocyte observations through HE, as well as immunohistochemistry of collagen type 2 and collagen type 1. The improvement was assessed with ICRS scores. The results obtained showed significant results between the control group and the bilayer scaffold. The three groups showed an increasing trend. The results emphasize the potential of bilayer scaffolds to be used in biomedical applications, especially in the repair of cartilage damage.

## 2. Materials and Methods

The animal trials have been approved by the Veterinary Ethics Committee of the Faculty of Veterinary Medicine, Universitas Airlangga (No. 2.KEH.181.12.2023), and were conducted in accordance with the guidelines of the Institutional Regulations for Animal Trials and the Basic Guidelines for Good Animal Experimental Behaviour.

### 2.1. Animal Experiments, Materials, and Instruments

Eighteen New Zealand female white rabbits from the animal laboratory, Faculty of Pharmacy, Universitas Airlangga, aged six to eight months, weighing between 2.0 and 2.5 kg. The materials which were used are chitosan (Sigma-Aldrich), BHA (PT. Inobi, Surabaya, Indonesia), sodium diclofenac (Elam Pharma Pvt. Ltd., Ankleshwar, Gujarat, India), glutaraldehyde (Sigma-Aldrich, Darmstadt, Germany), antibody collagen type 1 (Antibodies-online, Limerick PA, USA), collagen antibody type 2 (Antibodies-online, Limerick PA, USA), xylazine xyla (Interchemie, Venray, Netherlands), ketamine (PT. Dexa Medica, Cikarang, Indonesia), ampicillin (PT. Meiji, Jakarta Selatan, Indonesia), povidon (PT. Afi Farma, Kediri, Indonesia). While, the instruments which were used are freeze-dry (PT. Buchi, Tangerang, Indonesia), Syringe (PT. Bukit Bersemi Abadi, Medan, Indonesia), Analytical Stain Ohaus CP 214 (Sigma-Aldrich, Darmstadt, Germany), Drill Bosh GBM350 (PT. Bosch Rexroth, Surabaya, Indonesia), SEM (Inspect S-50, FEI, Japan), and FTIR (Perkin Elmer, MA, USA).

### 2.2. Preparation of the Scaffold

First layer: 500 mg of chitosan was dissolved in 10 mL of 1% acetic acid at 25°C. 500 mg of gelatin was dissolved in 3 mL aquadest at 50°C. The chitosan and gelatin solutions were mixed to form a homogenous solution. 1 mL of 2N NaOH was added to the solution until the pH became neutral. 10 mg sodium diclofenac was dissolved in 2 mL aquadest at 25°C. Then, the diclofenac sodium was added to the chitosan and gelatin mixture. 1 mL of 0.5% glutaraldehyde was added and then mixed [17].

Second layer: 500 mg of gelatin was dissolved in 3 mL aquadest at 50°C. Then, 1.5 grams of BHA was added to the gelatin solution and was mixed till homogenous. 500 mg of chitosan was dissolved in 10 mL of 1% acetic acid at 25°C and was added to the gelatin-BHA mixture. 1 mL of 2N NaOH was added to the solution until neutral. 1 mL of 0.5% glutaraldehyde was added and then was mixed [18].

Bilayer scaffold: Two cylindrical moulding instruments were prepared (each one 2 × 2 mm). Each layer suspension was inserted to the moulding. Both mouldings were stacked and frozen for a day, then freeze-dried for 2 × 24 hours at −50°C.

### 2.3. Characterization of the Scaffold

#### 2.3.1. FTIR Test

Functional characterization tests were carried out on the scaffold ingredients, namely, chitosan (Chi) powder, gelatin (Gel), bovine hydroxyapatite (BHA), and sodium diclofenac on the FTIR.

#### 2.3.2. Porosity Test

The porosity test is done using the fluid transfer method. Ethanol was used because it is easily absorbed in the scaffold without the presence of contraction and swelling. After freeze-drying, the scaffold was weighed as the dry weight of the scaffold (m1). Subsequently, the weight of the container and the ethanol was weighed as m2. After that, the scaffold was placed in a container containing the ethanol (m3). Then, it is soaked in 96% v/v ethanol for 48 hours. After 48 hours, the scaffold was removed, and the weight of the container and ethanol was weighed (m4). The porosity can be calculated using the following formula:

(1) $$\begin{array}{c} Porosity\;\left(\%\right)=\frac{\left(m3-m4-m1\right)}{\left(m2-m4\right)}\times 100\%. \end{array}$$

#### 2.3.3. Degradation Test

The degradation test was carried out by immersing the scaffold in a pH 7.4 PBS solution at 37°C in the incubator. A cubic device of 1 × 1 × 1 cm with 4 interconnected holes was used. Each layer of it in its input was prepared and dried using a freezer dryer. Scaffold bilayer in each test layer was obtained by immersion for 14 days, and the change of the mass was measured at intervals of 1 hour, 6 hours, 1 day, 3 days, 7 days, and 14 days [19].

#### 2.3.4. Compressive Test

In this test, the scaffold was given a load until the scaffold experienced distortion. This test used an autograph tool with a speed of 5 mm/minute. The scaffold was placed in the middle position of the tool table. The tool was turned on and set at a speed of 5 mm/minute, recording the diameter of the support and the height of the scaffold. The autograph will automatically stop and a force (N) will appear which can then be used to determine the compressive strength value.

#### 2.3.5. SEM and EDX

The test was carried out by cutting a sample of the scaffold with a width of 5 mm and a height of 3 mm. The scaffold was coated with gold using a sputter for 3 minutes. The sample that has been coated was inserted into the sample room and irradiated with an electron beam at a 1000× zoom and a voltage of 5 kV. The beam would be reflected and then detected by the sensing detector. The final result could be an image with an order of micron enlargement, which could be known as the size of the pores of the scaffold sample. This test was carried out by using SEM.

#### 2.3.6. Effectiveness of the In Vivo Scaffold

Eighteen New Zealand white rabbits were divided into control groups, the Chi-Gel-BHA scaffold group, and the bilayer scaffold group (*n* = 6). Anaesthesia was injected into the rabbit's hip intramuscularly. After 4 weeks, animals were sacrificed, and the specimens were collected. Rough injection, HE coloring, the immunohistochemical coloring of type II collagen, and type 1 collagen immunohistochemistry were performed to observe the cartilage.

#### 2.3.7. Analytical Method

GraphPad Prism 8 (GraphPad Software, Boston, USA) statistics software is used for analysis. The data are expressed as a mean ± SD. ICRS scores are stated as mean SD. The significance of the differences between groups was tested using a nonparametric test of the Kruskal–Wallis. Significance is considered to be *p* < 0.05.

## 3. Results and Discussion

Characteristics of bilayer scaffolds began with an FTIR examination, porosity test, degradation test, compressive strength test, and scanning electron microscope (SEM) test.

### 3.1. Bilayer Scaffold Formula

**Figure 1:**
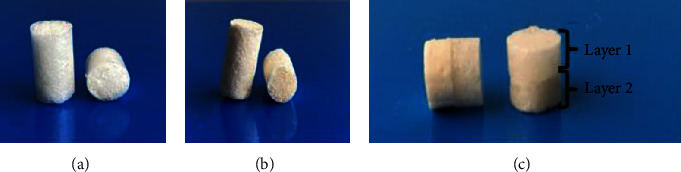
(a) First layer scaffold (Chi-Gel-ND), (b) second layer scaffold (Chi-Gel-BHA), and (c) bilayer scaffold.

The composition and description of the bilayer scaffold formula is as follows (Table 1 and Figure 1).

### 3.2. Fourier Transform Infrared (FTIR) Examination

FTIR is a spectroscopic method that uses infrared (IR) light to identify the functional groups of a compound. The spectrum results describe molecular absorption and transmission and create a fingerprint area of a sample. No molecular structure has the same IR spectrum. That reason makes IR spectroscopy more valuable in several types of analysis. Functional group characterization testing was carried out on scaffold materials, namely, chitosan powder, gelatin, BHA, and sodium diclofenac. Apart from that, testing was also carried out on the cartilage and subchondral layer scaffolds.

**Figure 2:**
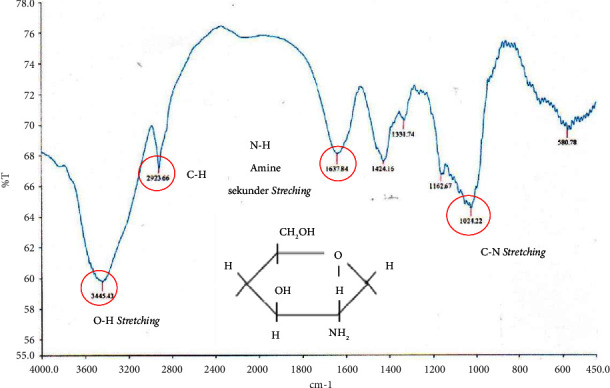
The FTIR spectrum of chitosan.

The FTIR spectrum that represents chitosan is the presence of a secondary NH amide group in the absorption area of 1637.84 cm^−1^, a C-N group in the absorption area of 1024.22 cm^−1^, and OH^−^ stretching at 3445.43 cm^−1^, as shown in Figure 2.

**Figure 3:**
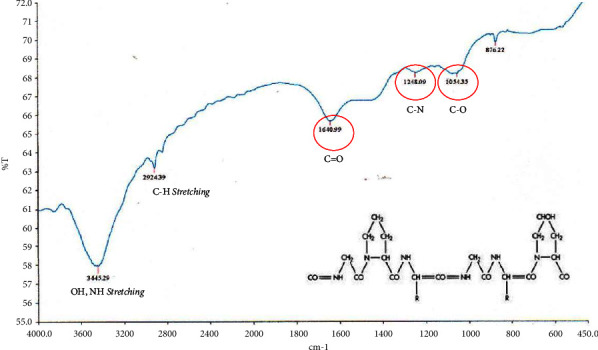
The FTIR spectrum of gelatin.

**Figure 4:**
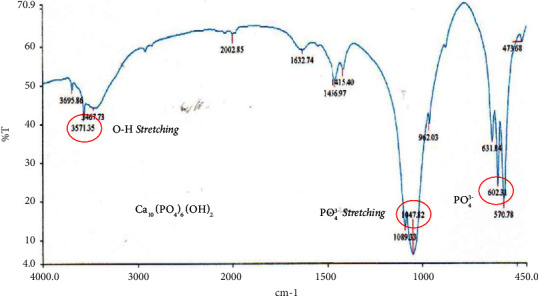
The FTIR spectrum of bovine hydroxyapatite.

The FTIR spectrum that represents gelatin as shown in Figure 3 is the presence of a specific proline group possessed by type I gelatin in the absorption area of 1340.82 cm^−1^, primary amine at 1640.99 cm^−1^, OH^−^ stretching at 3445.29 cm^−1^, and CH stretching at 2924.39 cm^−1^. The FTIR spectrum that represents BHA as shown in Figure 4 is the phosphate group (PO_4_^3-^) stretching in the absorption area of 1048.08 cm^−1^, (PO_4_^3−^) bending in the absorption area of 602.37 cm^−1^, and the hydroxyl group (OH^−^) in the absorption area of 3571 0.38 cm^−1^.

**Figure 5:**
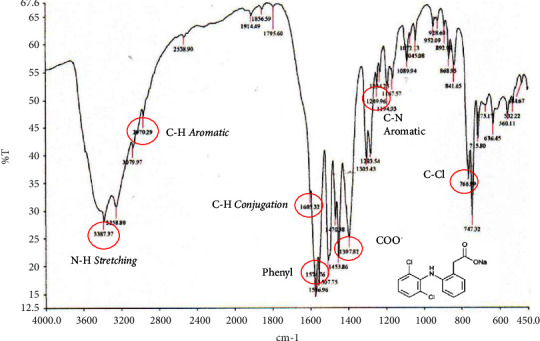
The FTIR spectrum of diclofenac sodium.

Diclofenac sodium as shown in Figure 5 has an absorption area of 1574.76 cm^−1^, which represents the phenyl group, conjugated CH=CH groups at 1603.32 cm^−1^, COO- groups at 1397.82 cm^−1^, C-Cl groups in the absorption area 766.99 cm^−1^, aromatic -CH group at 2970.29 cm^−1^, and secondary amine (-NH_2_) at 3387.37 cm^−1^.

**Figure 6:**
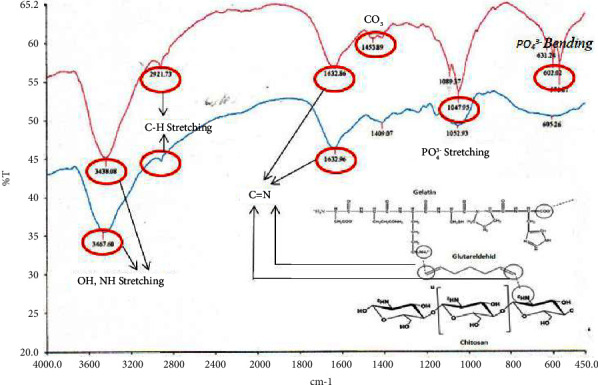
The FTIR spectrum of bilayer scaffold.

In the scaffold sample, a cross-link reaction was carried out to form a C=N (imine) bond, which was at an absorption of 1690 cm^−1^–1590 cm^−1^ [20]. The clusters that illustrate the cross-link reaction occurring in the first and second layers of the scaffold are shown in Figure 6. C=N (imine) bonds are formed in the absorption area of 1632.96 cm^−1^ for the first layer and 1632.86 cm^−1^ for the second layer. The aldehyde functional group (-CHO) in glutaraldehyde occurs in a cross-link reaction with the amino group (-NH_2_) in gelatin, chitosan, and diclofenac sodium.

The COO- group shifted from 1397.82 cm^−1^ to 1453.89 cm^−1^. Groups in the first and second layers, such as hydroxyl, are seen shifting from 3445.29 cm^−1^ to 3438.09 cm^−1^ and 3467.60 cm^−1^, respectively. The absorption for the P-O bond in the second layer, the PO_4_^3−^ group, is at an absorption of 1047.95 cm^−1^, shifted slightly from the previous 1047.82 cm^−1^. The C-N stretching group representing chitosan is seen in the absorption area of 1052.93 cm^−1^ and the aromatic C-N representing diclofenac sodium is seen in the absorption area of around 1240 cm^−1^.

### 3.3. Porosity Test

An ideal porous scaffold for clinical applications in subchondral tissue engineering must meet both structural and functional requirements. Scaffold layers that differ from one layer to another can mimic the natural gradient of subchondral tissues. After implantation, the scaffold can stimulate tissue regeneration and integration with the surrounding cartilage and subchondral bone [21]. Porosity and pore size have a significant influence on making three-dimensional (3D) scaffolds.

The percentage of porosity of the bilayer scaffolds, respectively, scaffold layer 1 (Chi-Gel-ND) is 87.00%, and the percentage of porosity of scaffold layer 2 (Chi-Gel-BHA) is 84.19% as shown in Tables 2 and 3. Bilayer scaffolds have superior porosity >80%. Scaffolds with porosity >80% will make it easier for cells to migrate, proliferate, and differentiate, thereby supporting cell growth [22, 23].

### 3.4. Degradation Test

**Figure 7:**
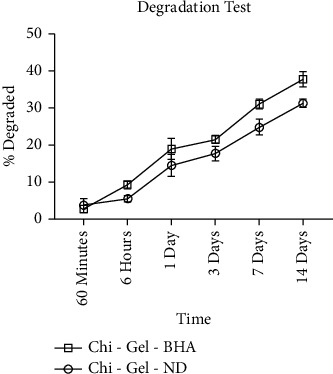
The degradation test of bilayer scaffold.

The degradation results of layer 1 are in order of time interval. These results vary at each time interval. It can be seen that BHA causes an increase in the percentage of scaffold degradation in the second layer, as shown in Figure 7.

### 3.5. Compressive Test

The compressive strength results of the bilayer scaffold tested for each layer are shown in Table 4.

### 3.6. Scanning Electron Microscope (SEM) Test

The examination was carried out using SEM, an electron microscope tool used to produce images of the sample surface with high resolution and very clear details. In SEM, a stream of electrons is directed toward the sample being observed, then reflected by the sample surface and directed toward the detector to produce an enlarged and clarified image. The main advantage of an SEM is its ability to magnify a sample millions of times, allowing users to see tiny details of the sample, such as surface structure, topography, and chemical composition. In SEM, the chemical composition analysis can be carried out using energy-dispersive X-ray spectroscopy (EDS) techniques or X-ray spectroscopy.

The inspection results show a clear boundary between layer one scaffold and layer two scaffold.

The results of measuring the pore diameter of the first layer scaffold were an average of 232.141 *μ*m, and the pore diameter of the second layer scaffold was an average of 98.678 *μ*m, as shown in Table 5. The recommended pore size conducive to scaffold cartilage regeneration is 30–100 *μ*m. In contrast, a larger pore size is needed to allow blood vessels to grow for bone tissues [24]. The pore diameter is the width of the pore holes formed in the scaffold. The effective pore size for bone growth is 100–600 *μ*m. If the pore size is too small, it will cause fewer cells to migrate and disrupt the process of nutrient exchange and the elimination of metabolic products. Meanwhile, if the pore size is too large, it can cause cells to separate more easily from the scaffold [25].

**Figure 8:**
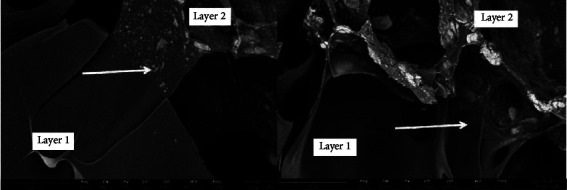
Scanning electron microscope (SEM) image of scaffold layer 1 and scaffold layer 2, arrows show the boundary between the two layers.

SEM images and pore widths are shown in Figure 8. The results of the EDX examination were carried out on both layers of the first layer scaffold and the second layer scaffold at several points to determine the compound content in the scaffold. They obtained a Ca/P ratio in layer 2 of 2.08 and 2.85.

### 3.7. Visual Observation of Defect Results on Day 28

**Figure 9:**
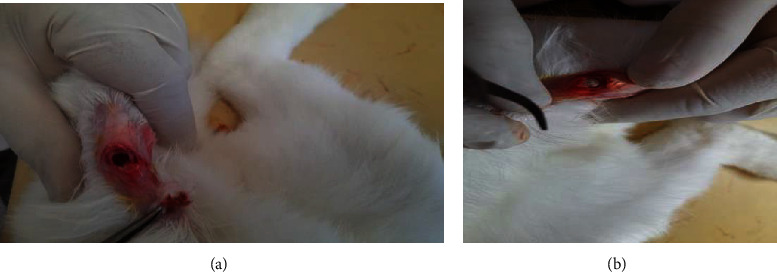
(a) The process of creating a defect in the rabbit femur trochlear (diameter 5 mm and 4 mm depth) and (b) implantation of the bilayer scaffold preparation.

The observation was carried out by making a defect model in the femoral trochlear with a diameter of 5 mm and a depth of 4 mm to the subchondral bone; then, implanting a bilayer scaffold and Chi-Gel-BHA scaffold in the defect that had been created is shown in Figure 9.

**Figure 10:**
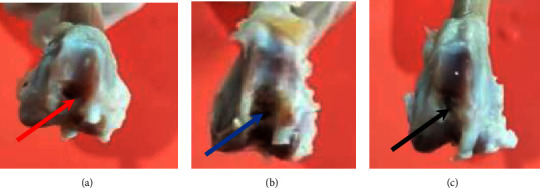
(a) Image of the defect without scaffold implant, (b) image of the defect with chitosan-gelatin-BHA, and (c) description of the defect with a bilayer scaffold.

On the 28th day after implantation, the test animals were sacrificed by injecting a lethal dose of ketamine (224 ± 4 mg/kg and 229 ± 5 mg/kg) intraperitoneally. Then, the right leg bone was tested macroscopically as shown in Figure 10. The effect of bilayer scaffold showed a smaller diameter of defect compared to control and chitosan-gelatin-BHA scaffold.

### 3.8. Histopathological Observation Results

The aim of observing HE staining is to determine the cartilage surface, matrix, distribution of chondrocytes, cell viability, subchondral bone, and calcified cartilage found in the defect area as shown in Table 6. This observation was carried out at 20 times and 40 times magnification to get a complete picture of the results of the defect in the femoral trochlear condyle. IHC results were figured out to determine the intensity and percentage of type I collagen and type II collagen as shown in Tables 7 and 8.

### 3.9. Data Analysis

Histologic assessment provides an important outcome measure of preclinical and clinical cartilage repair. In most histologic grading systems, readers grade the dominant tissue repair features on an ordinal scale of 0 to 3 or 4, which provides a semiquantitative and relatively rough estimate. The data were then analyzed using nonparametric statistical tests, which are Kruskal–Wallis tests.

Capillaries that have chondral defects are dissected and immediately subjected to microscopic examination. Macroscopic and microscopic assessments of cartilage healing were done by using the International Cartilage Repair Society (ICRS) as shown in Tables 9 and 10 [27]. For histological examination, bones were decalcified using EDTA and embedded in paraffin blocks. The tissue was cut into micrometer-thick sections sagittally and stained with hematoxylin-eosin (HE) and immunohistochemistry (IHC). The histological results are evaluated with the ICRS for histological grading as shown in Table 11 [26]. For macroscopic and histological examination, the scores were assessed by one clinical pathologist, the researcher, the main supervisor, and two research group colleagues.

**Figure 11:**
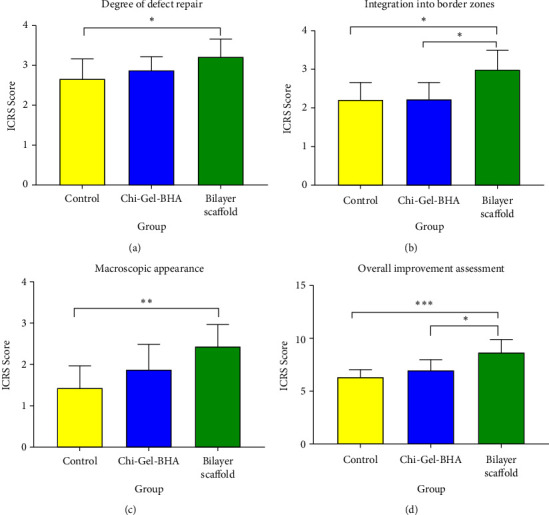
Evaluation of macroscopic cartilage healing based on ICSR score: (a) degree of defect repair, (b) integration into border zones, (c) macroscopic appearance, and (d) overall improvement assessment. ^∗^*p* < 0.05, ^∗∗^*p* < 0.01, and ^∗∗∗^*p* < 0.001.

The results of the macroscopic assessment based on ICRS and the overall improvement in bone repair on day 28 (Figure 11) can be categorized, namely, that the control received grade 3/not normal, the BHA scaffold received grade 3/abnormal, and the bilayer scaffold received grade 2/near normal with a value of 8.667 ± 1.225. The control and BHA scaffolds received the same grade. However, the average value was still greater for the BHA scaffold 7.000 ± 1.000 than the control 6.333 ± 0.7071.

**Figure 12:**
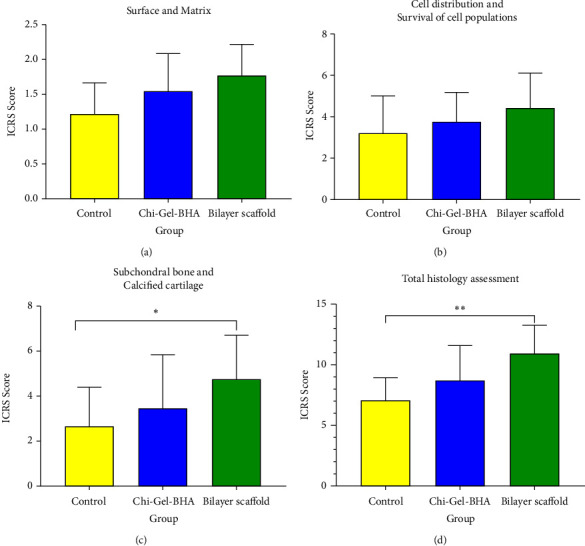
Histological evaluation of cartilage healing based on the ICSR score: (a) surface and matrix, (b) cell distribution and viability of cell populations, (c) calcified subchondral bone and cartilage, and (d) total histology assessment. ^∗^*p* < 0.05 and ^∗∗^*p* < 0.01.

The results of the histologic assessment were based on ICRS for 6 categories, namely, surface, matrix, cell distribution, cell population viability, subchondral bone, and cartilage mineralization. In descriptive data, the average score obtained is shown in Figure 12.

The results of the IHC examination showed that the intensity of type I collagen in the bilayer scaffold was higher than in the control group and lower than that in the Chi-Gel-BHA scaffold, although the percentage of type I collagen was lower than the control and Chi-Gel-BHA scaffolds. On the other hand, the intensity and percentage of collagen type II bilayer scaffolds are higher compared to the control group, and Chi-Gel-BHA scaffolds are shown in Table 12.

## 4. Discussion

A bilayer scaffold is a scaffold that is made to imitate the composition of cartilage and subchondral. The composition of the bilayer scaffold is composed of the first layer of chitosan-gelatin-diclofenac sodium and the second layer of chitosan-gelatin-BHA. Two different layers of a bilayer scaffold are suitable for use in cartilage/subchondral tissue engineering, which is expected to regenerate the bone simultaneously. In addition, the bilayer scaffold can maintain stability and good mechanical properties in the early stages of implantation. It can effectively avoid displacement and shifting of the cartilage layer.

Characterization of the bilayer scaffold formula was carried out for each layer. Porosity is one of the most important thing to be characterized. Through the porosity, the cells can exchange the nutrients, so it will keep surviving [28]. The results showed that the percentage of scaffold porosity layer 1 (Chi-Gel-ND) was 87.00%, and the percentage of scaffold porosity layer 2 (Chi-Gel-BHA) was 84.19%. Bilayer scaffolds have superior porosity >80%. Scaffolds with porosity >80% will make it easier for cells to migrate, proliferate, and differentiate, thus supporting cell growth [29]. The porous nature of the scaffold will facilitate cell development, transport body fluids, and support cells to proliferate and differentiate [23]. Meanwhile, for the Chi-Gel-BHA scaffold, which is intended as a substitute for trabecular (cancellous) bones, the porosity is >80%, which has a porosity of at least 70% [24]. In scaffolds, the cartilage layer has greater porosity in accordance with its role, following the cartilage structure, which is suitable for cell development and nutrient transport. Meanwhile, the subchondral scaffold layer has smaller porosity. It is expected to have better mechanical strength following its nature as trabecular bones.

In the degradation test, the results of each layer varied at each time interval. The materials used to make the bilayer scaffold were natural polymer materials, such as gelatin, chitosan, and the addition of glutaraldehyde for cross-links so that the degradation results could be regulated and controlled and could be used as a delivery agent for the drug sodium diclofenac. Regulated and controlled degradation is relevant to the formation of newly regenerated cartilage tissues, and the products released during degradation should be nontoxic to the body and easily eliminated [30]. Too high a degradation rate can cause the scaffold to break down, inhibiting mass transfer and leading to tissue necrosis. Meanwhile, a scaffold degradation rate that is too slow makes tissue regeneration slow because a fibrous capsule is formed, which will reduce the strength of the scaffold on body tissues [31].

The results of the compressive strength values for both layer 1 and layer 2 are still below this value range even though the addition of a cross-linking agent such as glutaraldehyde, which can form cross-linking bonds between the polymers that make up the scaffold and these bonds are expected to improve the mechanical strength of the scaffold [32]. Compressive strength results for the cartilage are usually in the range of 0.5 to 0.9 MPa [33], and the trabecular bone has a compressive strength ranging from 0.22 to 10.44 MPa, with an average value of 3.9 MPa [34]. The addition of chitosan to each layer can reduce compressive strength. Suppose the scaffold has a compressive strength value that is much lower than the compressive strength value of bone tissues. In that case, it will cause the scaffold to be unable to compensate for the pressure, which ultimately causes the scaffold to collapse. A compressive strength value that is greater than the implanted tissue will cause the scaffold to become very rigid and inflexible when pressure is applied, which can cause tissue necrosis [28]. The main limitation of chitosan is its low mechanical strength. It is reported that the compressive strength of chitosan scaffolds is very different, in the range of 0.0038–2.56 MPa and 0.059–0.125 MPa [35].

In the scanning electron microscope (SEM) test, the pore diameter of the first layer scaffold intended for the cartilage was an average of 232.141 *μ*m. At the same time, the porous sac with a size of 150–250 *μ*m best increased the expression and production of type II collagen and aggrecan, improving the formation and properties of the cartilage mechanics [36], and the pore diameter of the second layer scaffold for subchondral is an average of 98.678 *μ*m. The pore diameter is the width of the pore holes formed in the scaffold. The effective pore size for bone growth is 100–600 *μ*m. If the pore size is too small, it will cause fewer cells to migrate and disrupt the process of nutrient exchange and the elimination of metabolic products [37, 38].

Meanwhile, if the pore size is too large, it can cause cells to separate more easily from the scaffold [25]. A scaffold with a porosity of 90% and a pore diameter of 300 *μ*m should not cause an immune response or cytotoxicity. The scaffold is intended for cell colonization, transfer of proteins, and other developmental factors used for tissue construction and regeneration [37, 39].

The main and important components of human bones are hydroxyapatite, a form of the minerals calcium (Ca) and phosphorus (P), which provide stiffness to bones [40]. The results of the SEM EDX examination showed that the Ca/P ratio of the bilayer scaffold was 2.08 and 2.85. Changes in the bone Ca/P molar ratio range between 0.58 and 2.34 (fetal to adult) and, therefore, rarely reach the theoretical value of 1.67. The deviation, reported as the Ca/P mass ratio (weight ratio), ranges between 1.3 and 2.2 in adult human bones. In contrast, the theoretical Ca/P mass ratio value for hydroxyapatite is 2.15 [41].

The characterization results above showed that the bilayer scaffold can be applied to treat subchondral defects. Furthermore, it was formulated into a bilayer scaffold. Preclinical tests were carried out using the method of implanting the scaffold formula in the rabbit femoral trochlear bone hole with a diameter of 5 mm and a depth of 4 mm. If a cartilage injury only affects the superficial part (not through the subchondral bone tide marks), then usually there is no healing process because it does not cause bleeding and an inflammatory response. Chondrocytes close to the injury site may proliferate and synthesize the matrix but fail to repair at the injury site because they do not migrate to the injury site. In contrast, mesenchymal stem cells cannot penetrate the injury site. A layer of a new matrix may appear on the surface, but no significant repair process occurs. So, there are attempts to drill subchondral bones in articular cartilage injuries [42].

On macroscopic examination in Figure 11, the red arrow is a positive control/defect without a scaffold. It still shows that the defect hole has not closed even though the size of the defect has started to decrease. It still has clear boundaries between normal tissues and the defect after 28 days. Meanwhile, the direction of the blue arrow is the result of the Chi-Gel-BHA scaffold/monolayer scaffold. The hole is still visible in the defect, but it begins to be covered by a thin layer of fibrous tissues on day 28. For the bilayer scaffold, shown in the direction of the black arrow, the defect is almost closed. All of them have the proliferation of fibrous tissues, although they still show holes. However, they are smaller than the positive control, and the Chi-Gel-BHA scaffold and cartilage growth have not completely fused. Cartilage growth covered more than 80% of the defect in 8 weeks, and the defect was completely repaired at 12 weeks in rabbit cartilage using autologous rabbit synovial fluid-derived mesenchymal stem cells [43].

The macroscopic ICSR score consists of 4 categories, namely, the degree of cartilage repair, integration with the surrounding cartilage, macroscopic appearance, and overall assessment of repair. In the descriptive data, the mean scores in the three categories and the total ICSR score were significantly higher in the Chi-Gel-BHA scaffold treatment group and the bilayer scaffold treatment group compared with the control. Macroscopically, the ICSR score is in the categories of the healing level and macroscopic appearance. It was found that the ICSR score of the bilayer scaffold group was significantly higher compared to the control but not significantly higher compared to the Chi-Gel-BHA scaffold. However, there was a trend toward increasing the average score of the bilayer scaffold. The integration with the surrounding cartilage of the bilayer scaffold was significantly higher compared to the Chi-Gel-BHA scaffold. In the total ICSR score, the bilayer scaffold group was significantly higher than the Chi-Gel-BHA Scaffold (*p* < 0.05) and control (*p* < 0.001) as shown in Figure 12 by letter D. The total macroscopic ICRS score of the overall repair bone repair reflected that the score can be categorized that the control received grade 3/not normal, the BHA scaffold received grade 3/not normal, and the bilayer scaffold received grade 2/near normal with a value of 8.667 ± 1.225.

The control and BHA scaffolds received the exact grade. However, the average value was still greater for the BHA scaffold 7.000 ± 1.000 than the control 6.333 ± 0.707. The assessment of ICRS repair macroscopic examination of the surface repair of the cartilage tissue examined at 16 weeks compared to 8 weeks showed fewer and less obvious depressions bordering the surrounding normal articular cartilage, thus approaching normal using mesenchymal stem cells and hyaluronic acid hydrogel composite [44]. The articular surface defects of rabbit joints were filled with opaque white tissues with some irregular tissue plaques on the surface, and the defects showed at 8 weeks using cocultured cells combined with the PCL-HA scaffold [45]. Macroscopic ICRS assessment showed that bilayer scaffold implantation could accelerate the closure of cartilage/subchondral defects better than the positive control and BHA scaffolds. Bilayer osteochondral scaffolds showed much better osteochondral defect repair results in rabbit models using human adipose-derived stem cells (hADSCs) at week 8 [46].

The ICSR score on histological evaluation consists of 6 categories, namely, surface, matrix, cell distribution, cell population viability, subchondral bone, and cartilage mineralization categories. In descriptive data, the results of the histologic assessment based on ICRS on day 28 showed that the average score of the three combined categories and the total ICSR score were higher for the bilayer scaffold compared to the control group and the Chi-Gel-BHA scaffold. In the surface and matrix categories of histology, there were no significant differences in cell distribution and survival. However, there was a trend towards an increase in the average score of the bilayer scaffold compared to the control group and the Chi-Gel-BHA scaffold. In contrast, in the subchondral bone and calcified cartilage, the ICSR score was significantly higher in the bilayer scaffold group compared with the control (*p* < 0.05). In total histological scores, the score in the bilayer scaffold group was higher than the control (*p* < 0.01). There was no significant difference with the Chi-Gel-BHA scaffold group. However, the average score of the bilayer scaffold was higher than the Chi-Gel scaffold group. This value shows that implantation of a bilayer scaffold can accelerate the closure of cartilage/subchondral defects by increasing chondrocyte cells in the cartilage better than the positive control and similarly accelerates defect closure with the BHA scaffold.

The HE histology results from the positive control group showed that chondrocyte cell development was not complete and was still spreading. Even though soft tissues had formed, which was starting to cover the defect area, a suitable arrangement between the cartilage and the subchondral was not yet visible. The Chi-Gel-BHA scaffold group had tide marks visible but had not yet completely formed. However, there was visible chondrocyte growth spread between the cartilage and calcified cartilage. In contrast, the bilayer scaffold had clear tide marks and chondrocyte growth in the cartilage and calcified cartilage areas. However, holes were still visible, which may still have remnants of the bilayer scaffold. This tissue cannot withstand high pressure and is easily deformed. During the healing of the articular cartilage, chondrocyte proliferation occurs. The cells that are responsible for the healing process in the articular cartilage are unable to produce macromolecules such as proteoglycans and collagen in sufficient quantities to create a robust and adherent matrix such as normal articular cartilage [42]. Articular cartilage damage appears to be associated with decreased bone mineral and loss of subchondral bone tissues [47].

This IHC examination approach is related to the measured expression of the intensity and percentage of appearance of type I collagen and type II collagen from all groups. This expression depends on the cells responsible for the healing process in the articular cartilage must be able to produce macromolecules such as proteoglycans and collagen in sufficient quantities to create a strong, mutually adherent matrix such as normal articular cartilage [42]. The intensity and percentage of collagen type II bilayer scaffolds were higher compared to the control group and Chi-Gel-BHA scaffolds. This reason proves that a bilayer scaffold containing chitosan, which is similar to glycosaminoglycans, which is a cartilage ECM component, can increase chondrogenesis [48]. Chitosan-based scaffolds can be implanted for developing chondrocytes, providing a tailored microenvironment for native cells to attach, colonize, and repair damaged tissues [49]. Scaffolds containing chitosan and gelatin with glutaraldehyde can carry out the formation of the extracellular matrix and expansion of fibroblasts, which produce collagen, by supporting the adhesion, viability, proliferation, and osteogenic differentiation capacity of preosteoblasts [18].

The intensity of type I collagen in the bilayer scaffold was higher than that in the control group and lower than that in the Chi-Gel-BHA scaffold, although the percentage of type I collagen was lower than that in the control and Chi-Gel-BHA scaffolds. Cartilage wound healing is a temporary balance between the deposition of type I collagen in the form of scar tissues and repair through the expression of type II collagen and proteoglycans, having a growth-promoting effect on cartilage [50]. The lower percentage of type I collagen in the bilayer scaffold indicates that the bone healing process in the subchondral has gone through a period (4 weeks), and it has become a soft callus. Pitch angle (PA) analysis of type I and II collagen showed that at the fracture healing stage after 4 weeks, the deep reduction in mean PA was consistent with the results obtained for control bone tissues, indicating that the PA peptide value of type I collagen (which dominates the final stage of bone tissue healing) is lower than type II collagen (which dominates the early stages of the cartilage or callus formation) [51].

Based on the results obtained, cartilage repair in the bilayer scaffold group containing diclofenac was better than the other two groups. This indicates that sodium diclofenac has a role in cartilage repair. The inflammatory response can inhibit cartilage repair in tissue engineering. Therefore, this response needs to be suppressed to obtain better regeneration results. Diclofenac can inhibit the production of PGE2 and nitrite through the cyclooxygenase-2 (COX-2) signalling pathway. The release of diclofenac from the scaffold has the potential to reduce neutrophils and macrophages at the site of injury, thereby reducing the inflammatory response.

## 5. Conclusions

Natural scaffolds are characterized by high bioactivity, biocompatibility, and biodegradability of nontoxic components. Due to its composition of natural materials, such as bovine hydroxyapatite, this scaffold is similar to the actual tissue, which means its presence creates an ideal environment for cells. Thus, the main advantage of natural polymers, such as chitosan and gelatin are their similarity to cartilage ECM components. Their presence precisely stimulates chondrogenesis and maintenance of chondrocyte cellular phenotype. These results show that the subchondral soft callus process was formed more quickly using the bilayer scaffold compared to the control and Chi-Gel-BHA scaffolds shown by the increasing value of ICRS. Diclofenac on scaffold has shown to accelerate cartilage regeneration by suppressing the inflammatory response through the COX2. Therefore, bilayer scaffolds can be used as an alternative allograft product for the treatment of cartilage and subchondral defects.

## Figures and Tables

**Table 1:** The composition of the bilayer scaffold.

First layer		Second layer	
Materials	Quantity	Materials	Quantity
Chitosan	0.5 g	Chitosan	0.5 g
Gelatin	0.5 g	Gelatin	0.5 g
Sodium diclofenac (ND) 1%	10 mg	Bovine hydroxyapatite	10 mg
Acetic acid 1%	10 mL	Acetic acid 1%	10 mL
Aquadest	5 mL	Aquadest	5 mL
Glutaraldehyde 0.5%	1 mL	Glutaraldehyde 0.5%	1 mL
NaOH 2N 1	1 mL	NaOH 2N	1 mL

**Table 2:** Percentage of porosity first layer scaffold (Chi-Gel-ND).

M1	M2	M3	M4	% porosity
0.0139	4.4613	4.4653	4.3663	89.58
0.0170	4.4205	4.4185	4.2949	84.87
0.0180	4.5457	4.5519	4.4695	84.51
0.0151	4.3338	4.3371	4.2215	89.49
0.0151	4.6656	4.6665	4.5601	86.54
Average				87.00 ± 2.44

**Table 3:** Percentage of porosity second layer scaffold (Chi-Gel-BHA).

M1	M2	M3	M4	% porosity
0.0487	4.6483	4.6857	4.5625	86.83
0.0497	4.7606	4.7963	4.6830	81.96
0.0481	4.6380	4.6661	4.5338	80.81
0.0499	4.7071	4.7486	4.6462	86.21
0.0474	4.6593	4.6942	4.5750	85.17
Average				84.20 ± 2.66

**Table 4:** The compressive strength of bilayer scaffold.

No	*σ* (MPa)	
	Layer 1	Layer 2
1	0.084	0.335
2	0.078	0.364
3	0.115	0.396
Average	0.093 ± 0.020	0.365 ± 0.030

**Table 5:** The diameter of the scaffold pore using scanning electron microscope (SEM).

Layer	Pore diameter
Layer 1	232.14 ± 23.29
Layer 2	98.68 ± 25.18

Data are shown as mean ± SD.

**Table 6:** Image of HE staining on day 28.

	20 x	40 x	400 x
Defect	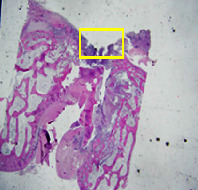	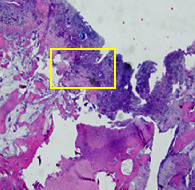	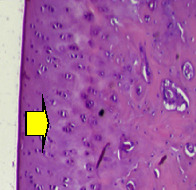

Chi-Gel-BHA	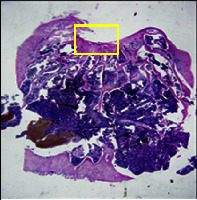	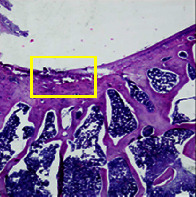	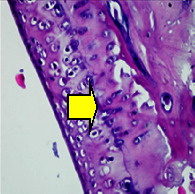

Bilayer scaffold	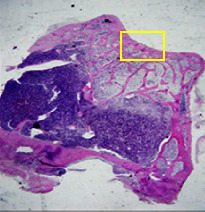	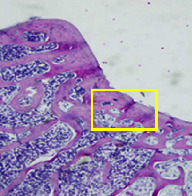	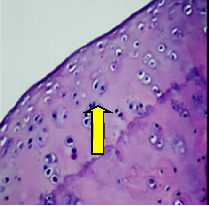

The yellow arrow means chondrocyte.

**Table 7:** IHC staining of type I collagen on day 28.

	20 x	40 x	400 x
Defect	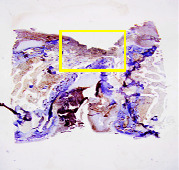	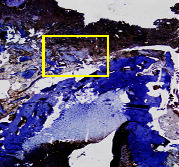	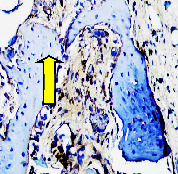

Chi-Gel-BHA	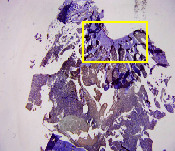	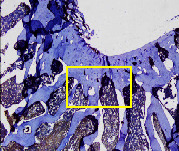	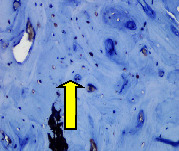

Bilayer scaffold	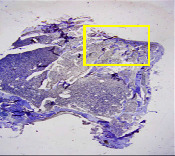	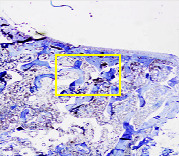	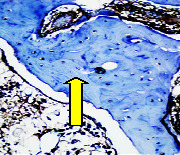

**Table 8:** IHC staining of type II collagen on day 28.

	20 x	40 x	400 x
Defect	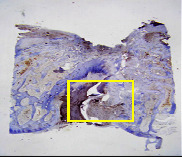	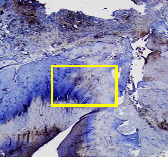	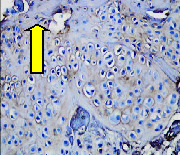

Chi-Gel-BHA	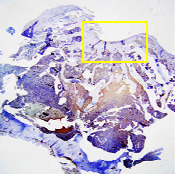	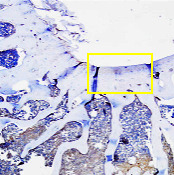	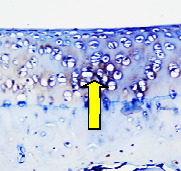

Bilayer scaffold	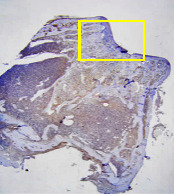	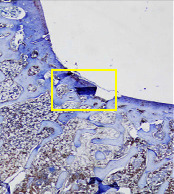	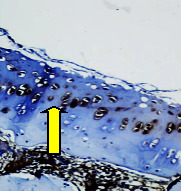

**Table 9:** Degree description ICRS assessment [26].

Level	Description	Explanation
Degree 0	Normal	—
Degree 1	Near normal	Superficial lesions. Soft indentations and/or shallow cracks and crevices
Degree 2	Abnormal	The lesion extends to <50% of the cartilage depth
Degree 3	Very abnormal	Cartilage defects extend to >50% of the cartilage depth and into the calcified layer
Degree 4	Very abnormal	Osteochondral injury, the lesion extends to the subchondral bone plate or deeper into its trabecular bone

**Table 10:** Macroscopic evaluation of cartilage repair using ICRS.

Category		Value
Degree of defect repair	Aligned with the surrounding cartilage	4
	75% improvement in defect depth	3
	50% repair of defect depth	2
	25% improvement in defect depth	1
	0% repair of defect depth	0

Integration into border zones	Complete integration with surrounding cartilage	4
	Limiting limit <1 mm	3
	¾ integrated grafts, ¼ with prominent edges >1 mm wide	2
	½ of the graft was integrated with the surrounding cartilage, ½ with a prominent margin of >1 mm	1
	From no contact to ¼ of the graft integrated with the surrounding cartilage	0

Macroscopic appearance	Smooth surface intact	4
	Pictorial surface	3
	Scattered small cracks or fissures	2
	Several, small or few but large gaps	1
	Complete degeneration of the grafted area	0

Overall improvement assessment	Grade 1: normal	12
	Grade 2: close to normal	8–11
	Grade 3: abnormal	4–7
	Grade 4: very abnormal	1–3

**Table 11:** Histological evaluation of cartilage repair using ICRS.

Histological appearance		Score
Surface	Smooth/continuous	3
	Discontinuity/irregularity	0

Matrix	Hyaline	3
	Mixed: hyaline/fibrocartilage	2
	Fibrocartilage	1
	Fibrous tissue	0

Cell distribution	Columned	3
	Mix/cluster columns	2
	Clusters	1

Survival of cell populations	Very worthy	3
	Worth it in part	1
	<10% worth it	0

Subchondral bone	Normal	3
	Increased remodeling	2
	Bone/granulation tissue necrosis	1
	Separated/broken/callus at the base	0

Calcified cartilage	Normal	3
	Abnormal/abnormal location	0

**Table 12:** Average results of IHC assessment of collagen type I and collagen type II.

Group	Collagen type I		Collagen type II	
	Intensity	Percentage	Intensity	Percentage
Control	2.00 ± 1.00	2.33 ± 1.20	2.33 ± 1.20	2.33 ± 1.45
Scaffold Chi-Gel-BHA	3.00 ± 0.00	3.50 ± 0.50	1.50 ± 1.50	0.50 ± 0.50
Scaffold bilayer	2.33 ± 1.20	1.67 ± 0.88	2.67 ± 1.45	3.00 ± 1.53

## Data Availability

The data that support the findings of this study are available from the corresponding author upon reasonable request.
